# IL28B and IL10R −1087 polymorphisms are protective for chronic genotype 1 HCV infection and predictors of response to interferon-based therapy in an East-Central European cohort

**DOI:** 10.1186/1756-0500-7-12

**Published:** 2014-01-08

**Authors:** Alajos Pár, Gabriella Pár, István Tornai, Ferenc Szalay, Dalma Várszegi, Edit Fráter, Mária Papp, Gabriella Lengyel, János Fehér, Márta Varga, Judit Gervain, János Schuller, Zsuzsanna Nemes, Zoltán Péterfi, Anna Tusnádi, Béla Hunyady, Attila Haragh, Zsolt Szinku, Áron Vincze, László Szereday, Péter Kisfali, Béla Melegh

**Affiliations:** 1 First Department of Medicine, University of Pécs, Rákóczi u. 2, 7623 Pécs, Hungary; 2 Second Department of Medicine, University of Debrecen, 4012 Debrecen, Hungary; 3 First Department of Medicine, Semmelweis University, 1082 Budapest, Hungary; 4 Department of Dermatology, University of Pécs, 7627 Pécs, Hungary; 5 Second Department of Medicine, Semmelweis University, 1088 Budapest, Hungary; 6 Réthy Pál Hospital, 5600 Békéscsaba, Hungary; 7 Szent György Hospital, 8000 Székesfehérvár, Hungary; 8 United Szent István and Szent László Hospital, 1097 Budapest, Hungary; 9 Hetényi Géza Hospital, 5004 Szolnok, Hungary; 10 Kaposi Mór Teaching Hospital, 7400 Kaposvár, Hungary; 11 Department of Medical Microbiology and Immunology, University of Pécs, 7624 Pécs, Hungary; 12 Department of Medical Genetics, University of Pécs, 7624 Pécs, Hungary

**Keywords:** Genetic polymorphism, Hepatitis C virus, Interferon, Interleukin-28B, Interleukin-10

## Abstract

**Background:**

Previous studies have shown that single nucleotide polymorphisms (SNP) in IL28B and IL10R are associated with sustained virological response (SVR) in chronic hepatitis C patients treated with pegilated interferon plus ribavirin (P/R). The present study extends our earlier investigations on a large East-Central European cohort. The allele frequencies of IL28B and IL10R in genotype 1 HCV infection were compared with that of healthy controls for the purpose of examining the relationship between the polymorphisms and the SVR to P/R treatment.

**Methods:**

A total of 748 chronic HCV1 infected patients (365 male, 383 female; 18–82 years) and 105 voluntary blood donors as controls were enrolled. Four hundred and twenty HCV patients were treated with P/R for 24–72 weeks, out of them 195 (46.4%) achieved SVR. The IL28 rs12979860 SNP was determined using Custom Taqman SNP Genotyping Assays. The IL10R −1087 (also known as IL10R −1082 (rs1800896) promoter region SNP was determined by RT-PCR and restriction fragment length polymorphism analysis.

**Results:**

The IL28B CC genotype occurred with lower frequency in HCV patients than in controls (26.1% vs 51.4%, p<0.001). P/R treated patients with the IL28B CC genotype achieved higher SVR rate, as compared to patients with CT (58.6% vs 40.8%, p=0.002). The prevalence of IL10R −1087 GG genotype was lower in patients than in controls (31.8 % vs 52.2%, p<0.001). Among patients achieving SVR, the IL10R −1087 GG genotype occurred with higher frequency than the AA (32.0% vs 17.4%, p=0.013). The IL28B T allele plus IL10R A allele combination was found with higher prevalence in patients than in controls (52% vs 20.7%, p<0.001). The IL28B CC plus IL10R A allele combination occurred with higher frequency among patients with SVR than in non-responders (21.3% vs 12.8%, p=0.026). Both the IL28B CC plus IL10R GG and the IL28B CC plus IL10R A allele combinations occurred with lower frequency in patients than in controls.

**Conclusions:**

In our HCV1 patients, both the IL28B CC and IL10R GG genotypes are associated with clearance of HCV. Moreover, distinct IL28B and IL10R allele combinations appear to be protective against chronic HCV1 infection and predictors of response to P/R therapy.

## Background

Hepatitis C virus (HCV) is a major global health problem: currently 170 million subjects are suffering from HCV infection worldwide [1,2]. The outcome of HCV infection ranges from spontaneous viral clearance to hepatitis, cirrhosis and hepatocellular carcinoma. Some individuals have rapidly progressive liver disease, while others remain symptom-free virus carriers. The exact causes of the different disease courses are not known. Immune mechanisms, as well as environmental factors are responsible for the various HCV-related events [3]. As genetic factors controlling the host immune response play a pivotal role in HCV infection, a genetic predisposition may also be crucial [4-6]. *Cytokines* are among the predominant mechanisms of host defense against infection; they induce inflammatory response that often leads to tissue injury, but also serve as antiviral effectors. Moreover, cytokine synthesis capacity has a significant *genetic component*. This explains why differences exist between individuals in their ability to produce cytokines, a fact which may also be due to single-nucleotide polymorphisms (SNPs) within the coding regions of cytokine genes. Cytokine genes are polymorphic and certain SNPs modify the cytokine production and affect the host immune response. In HCV infection, secretion of an inappropriate amount of cytokines may be associated with chronicity or resistance to interferon (IFN) treatment [7].

The *interleukin-28B (IL-28B*) – also referred to as interferonλ-3 (IFNλ-3) [8-12] – was shown to be involved in the control of HCV infection. The genetic polymorphisms of the encoding IL28B gene region determine the spontaneous and treatment induced clearance of genotype 1 HCV (HCV1) [13-15].

IL-28B is expressed by peripheral blood mononuclear cells, dendritic cells, and hepatocytes upon infection with viruses or stimulation with double-stranded RNA. IL28B in turn activates signal transduction through the JAK-STAT pathway, exerts antiviral activity and has an impact on natural clearance of HCV. IL-28B exhibits fewer IFN-like adverse effects because IL-28B receptors are expressed on a limited number of cell types. The receptor for IL-28 is composed of a unique IL-28R-α chain that pairs with the IL-10R-β chain [10,11]. IL28B gene on human chromosome 19q was discovered using genomic screening process in which the entire human genome was scanned for putative functional variants [8,9].

Ge et al. [12] identified first a SNP (rs12979860) located only 3 kilobases upstream from the IL28B gene, that encodes IL-28B. They applied genome-wide association study (GWAS), retrospectively analysed samples of 1,137 HCV infected individuals participating in a clinical trial with pegylated interferon plus ribavirin (P/R) therapy, and found that the high IL-28B production CC genotype was associated with approximately a threefold greater rate of SVR to P/R when compared with the TT genotype. This finding was confirmed by others [13,14]. At the same time it was also shown that the frequency of the CC genotype was significantly lower in HCV patients when compared to matched controls; thus, suggesting that this variant may be linked to a higher rate of natural clearance of HCV. This hypothesis was soon strengthened by Thomas et al., who reported that the IL28B CC genotype enhanced the spontaneous resolution rate of HCV infection [15]. Thus, it became evident that HCV patients who harbor the CC genotype at rs12979860 are more prone to respond to P/R treatment and to clear the virus than patients who do not possess this genetic polymorphism.

*The standard of care (SOC) treatment* for HCV infection is peginterferon plus ribavirin (P/R) therapy of 24–72 month duration, depending on HCV genotype, pre-treatment viral load and viral kinetic. This type of treatment may result in sustained virological response (undetectable HCV RNA after 24 weeks of the end of therapy) in approximately 40–45% of patients infected by genotype 1 HCV (HCV1) the most common genotype in Europe. In the past 2 years, new compounds, “*direct acting antivirals” (DAAs*) have also been developed such as HCV protease- and polymerase inhibitors, that significantly enhance SVR compared with SOC even to 68–74% in treatment-naïve HCV1 individuals [16].

Since there are conflicting data on the role of the regulatory Th2 cytokine *interleukin-10* (IL-10) in HCV infection [17-20], the polymorphism of the IL10R gene has also been examined. In our preliminary studies, the first from an East-Central European country, we reported that not only IL28B but also IL10R −1087 (also known as IL10R-1082) genetic variants may play a role in HCV infection [21,22].

Interleukin-10 (IL-10), produced by monocytes, macrophages and T cells, inhibits both the activation of CD4+ T-helper cells and the function of cytotoxic CD8 + T, NK and antigen-presenting cells, and also modulates hepatic stellate cell collagen synthesis. IL-10 plays a regulatory role in immune reaction and suppresses inflammatory responses by inhibiting the production of pro-inflammatory cytokines. Its effect is mediated through the IL-10 receptor (IL-10R), which is a heterodimer that consists of both IL-10R1 required for binding, and IL-10R2 required for signaling. Increased IL-10 production was reported in association with the -1082G, -819C, -592GCC IL-10 promoter (ATA) haplotype [23,24]. If macrophages secrete large amount of IL-10, it may decrease circulating TFN-α and IL-6 levels, thus, reducing their harmful effects. A higher frequency of IL10 -1082 GG genotype was found in older healthy controls than in patients with myocardiac infarction; high IL-10 production was protective for longevity [25].

The present study extends our previous investigations on a large East-Central European cohort in Hungary. The allele frequencies of IL28B and IL10R −1087 in HCV1 infection were compared with that of healthy controls for the purpose of examining the relationship between the polymorphisms and the response to PEG-IFN and ribavirin (P/R) treatment. We find that in HCV1-infected East-Central European patients, both the IL28B CC and IL10R GG genotypes are associated with clearance of HCV. Moreover, distinct IL28B and IL10R allele combinations appear to be protective against chronic HCV1 infection and predictors of response to P/R therapy.

## Methods

### Ethics

This work has been carried out in accordance with the guidelines of the 1975 Declaration of Helsinki and has been approved by both the National Ethics Committee (ETT TUKEB Budapest, No 490/010) and the Regional Ethics Committee at the University of Pécs.

All the patients agreed to participate in the study, and provided written informed consent.

### Patients

A total of 748 HCV1 infected patients (365 men, 383 female, ranging in age from 18 to 82 years, mean 54 ± 10 years) were enrolled. All subjects were inpatients in 10 Hungarian city hospitals or university clinics between January 2007 and December 2009.

The diagnosis of chronic hepatitis C was based on persistent elevation of serum alanine aminotransferase (ALT) levels >6 months in the presence of anti-HCV by ELISA and HCV RNA by real-time reverse transcriptase-polymerase chain reaction (RT-PCR). Quantitation of plasma HCV RNA was carried out using Cobas Amplicor 2.0 (Hoffmann-La Roche Inc.). HCV genotype was determined by a genotype specific probe based assay in the 5′ untranslated region (LiPA; Innogentics, Ghent, Belgium). (In Hungary, >95% of HCV patients have been infected with HCV genotype 1. In Eastern European countries the prevalence of genotype 1 ranges between 57% and 99% [26]). In 70% of our patients percutaneous liver biopsy was performed.

Of the 748 HCV1 patients, 420 were treated with pegylated interferon alfa 2a/2b (Pegasys, Hoffmann-La Roche Inc./Pegintron, SP Labo N.V. Belgium) 135–180 μg/1.0-1.5 μg/kg subcutaneously per week, and ribavirin (Copegus, Hoffmann-La Roche Inc./Rebetol, SP Labo N.V. Belgium) 1000–1200 mg orally per day for 24–72 weeks, then followed up for 24 weeks.

Subjects who had undetectable HCV RNA levels following 24 weeks of therapy were designated as sustained virological response (*SVR*), undetectable serum HCV RNA at week 4 after starting treatment as rapid virological response (*RVR*), undetectable HCV RNA at week 12 after starting therapy as early virological response (EVR). In our paper, patients who did not achieve SVR were regarded as “non-responders” (*non-SVR*).

One hundred and five healthy individuals (64 men, 41 female, mean age 45 ± 3 years), who were consecutive voluntary blood donors with normal liver function tests and negative for HBV, HCV and HIV serology, served as controls.

### Genotyping

DNA was isolated from peripheral blood by a standard desalting method. The **IL28B** rs12979860 SNP was determined using Custom Taqman SNP Genotyping Assays (Applied Biosystems, Life Technologies, Foster, CA, USA). The **IL10R −1087** (also known as IL10R −1082) *(rs1800896)* promoter region was required for the formation of the EcoNI recognition sequence 5′-AAGACAACACTACTAAGGCT-3′; the lower primer was 5′-TAAATATCCTCAAAGTTCC-3′. After cutting the amplified product (584 bp) by EcoNI, homozygote GG was identified by two fragments 315 and 279 bp, while heterozygote AG had 310, 280, 252 and 28 bp fragments and homozygote AA had 310, 252 and 28 bp fragments [27].

### Statistical analysis

Statistical analysis was carried out using the SPSS 16.0 for Windows (SPSS Inc., Chicago, IL, USA). The allele and genotype frequencies were compared with Pearson’s χ2 test. Binary logistic regression analysis was performed to observe the individual contributions of the polymorphisms. A p value of <0.05 was considered significant.

## Results

Of 420 peginterferon and ribavirin (P/R) treated patients 195 (46.4%) achieved SVR. Patients with RVR (20% of treated) achieved a 86% SVR rate, while those with EVR (55% of treated) showed a 65% SVR rate.

As shown in Table 1, when comparing the **IL28B** genotype frequencies between groups of healthy controls and patients, the CC genotype occurred with lower frequency in HCV patients than in controls, thus, suggesting its protective effect against chronic hepatitits C. On the other hand, CT heterozygosity and T alleles were more prevalent in patients, and may, therefore, convey susceptibility for the disease.

**Table 1:** Prevalence of IL28B genotypes in Hungarian HCV patients and healthy controls

**IL28B genotype**	**HCV1 (n = 748)**	**Controls (n = 105)**	**OR**
**CC**	195 (26.1%)	54 (51.4%)	0.333 (0.22 - 0.505)
			P < 0.001
**CT**	411 (54.9%)	39 (37.1%)	2.064 (1.354 - 3.145)
			p = 0.001
**C allele**	606 (81.02%)	93 (88.6%)	0.551 (0.294-1.032)
			p = 0.059
**TT**	142 (19.0%)	12 (11.4%)	1.816 (0.969 – 3.404)
			p = 0.059
**T allele (non-CC)**	553 (73.9%)	51 (48.6%)	3.003 (1.981 – 4.552)
			P < 0.001

As shown in Table 2, subjects that received P/R therapy and who had the IL28B CC genotype achieved higher rates of SVR than subjects who had CT genotype (58.6% vs 40.8%) (OR 2.057, 95% CI: 1.305-3.058, p = 0.002), or those carrying the T allele (41.8%, OR 1.976, 95% CI: 1.263-3.058, p = 0.002).

**Table 2:** Sustained virological response (SVR) rate stratified by IL28B genotypes in Hungarian HCV patients

	**Treated**	**SVR**	
**IL28B genotype**	**n**	**Number of patients**	**Percentage**
**CC**	116	68	58.6%
**CT**	228	93	40.8%
**TT**	76	34	44. 7%
**T allele (non-CC)**	304	127	41.8%

As shown in Table 3, IL10R GG genotype occurred with lower frequency in patients (31.8%) than in controls (52.2%) (OR: 0.428, p < 0.001). The prevalence of IL10R AA allele was 68.15% in patients and 47.8% in healthy individuals (OR: 2.335, p < 0.001).

**Table 3:** Prevalence of IL10R −1087 genotypes in HCV patients and healthy controls

**IL10R −1087 Genotype**	**HCV1 (n = 672)**	**Controls (n = 92)**	**OR**
**GG**	214 (31.8)	48 (52.2%)	0.428 (0.276 - 0.665)
			p < 0.001
**GA**	333 (49.6%)	32 (34.8%)	1.842 (1.169 – 2.903)
			p = 0.008
**G allele**	547 (81.4%)	80 (87.0%)	0.656 (0.347-1.241)
			p = 0.192
**AA**	125 (18.6%)	12 (13.0%)	1.523 (0.806 – 2.881)
			p = 0.192
**A allele (non-GG)**	458 (68.15%)	44 (47.8%)	2.335 (1.504 – 3.625)
			p < 0.001

Among P/R treated patients with SVR, the IL10R **GG** genotype occurred with higher frequency than the **AA** genotype, (57/178, 32.0% vs 31/178, 17.4%, (OR: 1.84, 95% CI 1.13-2.98, p = 0.013).

The SVR rate in patients with IL10R **GG** genotype was **42.2%** (59/125); in those with GA **47.4%** (88/178) and in **AA** patients **39.7%** (31/78). HCV patients with **A** allele (non-GG genotype) achieved SVR in **46.4%** (119/256).

### Genotype combinations

#### Healthy controls and HCV1 patients

The prevalence of the double wild type combination of the **IL28B CC plus IL10R GG** was lower (9.2%) in HCV patients than in healthy controls (23.9%) (OR 0.322, p < 0.001).

The **IL28B CC plus IL10R A** allele combination likewise occurred with lower frequency in patients (16.1%) when compared with controls (27.2,%, OR 0.515, p = 0.009). Both combinations suggest a protective effect against chronic HCV infection.

The prevalence of **IL28B T allele plus IL10R A** allele combination was higher in patients (52.4%) than in healthy controls (20.7%), thus, leading to a more than fourfold risk increase of the disease (OR 4.231, p < 0.001) (Table 4).

**Table 4:** IL28B and IL10R −1087 genotype combinations in the different study groups

			**IL28B genotype**			
			**CC**	**CC**	**T allele**	**T allele**
**IL10R genotype**	**HCV n = 664**	GG	61 (9.2%)	OR:0.322 (0.186-0.556)	148 (22.3%)	0.728 (0.446-1.188)
		A allele	107 (16.1%)	p < 0.001	348 (52.4%)	p = 0.202
	**Controls n = 92**	GG	22 (23.9%)	OR:0.515 (0.311-0.852)	26 (28.3%)	OR:4.231 (2.497-7.169)
		A allele	25 (27.2%)	p = 0.009	19 (20.7%)	p < 0.001
	**SVR n = 178**	GG	22 (12.4%)	OR:1.648 (0.837-3.247)	37 (20.8%)	OR:0.803 (0.496-1.301)
		A allele	38 (21.3%)	p = 0.146	81 (45.6%)	p = 0.372
	**Non-responders n = 203**	GG	16 (7.9%)	OR: 1.848 (1.070-3.190)	50 (24.6%)	OR:0.692 (0.462-1.037)
		A allele	26 (12.8%)	p = 0.026	(54.7%)111	p = 0.074

Prevalence of IL28B CC plus IL10R GG in HCV patients vs. controls OR: 0.322.

IL28B CC plus IL10R A allele OR: 0.515.

IL28B T allele plus IL10R A allele OR: 4.231.

Prevalence of IL28B CC plus IL10R A allele in pts with SVR vs. non-responders OR:1.848.

### Comparison of subjects with sustained virologic response (SVR) versus non-responder (non-SVR) patients

The **IL28B CC plus IL10R −1087 AA** allele combination was found with higher frequency among patients achieving SVR (21.3%), than in non-responders (12.8%, OR 1.848, 95% CI 1.070-3.190, p = 0.026). In patients with SVR, the prevalence of the **IL28B CC plus IL10R GG** combination was 12.4%, whereas in non-responders it was 7.9% (OR 1.648, NS) (Table 4).

Cumulatively, the data suggest that SNPs in both IL28B and the IL-10 promoter region, and specific combinations thereof, are associated with protection against HCV infection and differential responsiveness to IFN therapy.

## Discussion

Previous studies have shown that IL28B SNP at the polymorphic site rs129798060 with the CC genotype, and IL10 receptor SNP at −1082 with the G allele are associated with susceptibility to chronic hepatitis C virus infection and response to combined antiviral therapy [12-14,17,28,29]. In our cohort of Hungarian patients, we evaluated these SNPs in a total of 748 patients infected with genotype 1 HCV from 10 centers throughout Hungary. To our knowledge, this is largest study of its type on HCV-infected subjects from East-Central Europe. Our results show that polymorphisms of both IL28B and IL10R genes play a role in the outcome of chronic hepatitis C in a new cohort. We were able to demonstrate that IL28B CC genotype is a strong predictor of SVR in patients treated with P/R, and protects against the disease. The current study on a large cohort confirms the results of our earlier studies [21,22]. This genetic variant of IL28B may be associated with an elevated production of IL-28B (IFNλ-3) but this has not been found in all studies [12].

Our results are in accordance with the previous findings [12-14]. The frequency of IL28B CC in HCV1 patients was lower than in healthy controls, this fact may mean that individuals with IL28B CC genotype are protected against chronic hepatitis C. Patients with IL28 CC genotype may have a high likelihood of SVR and possibly need only the standard of care P/R treatment, particularly if they have other positive predictors: low viral load, young age, female, short duration of infection, absence of cirrhosis and low BMI. Under such circumstances, an IL28B CC will not only predict SVR but also motivate both the patient and the doctor, serving to enhance adherence as well However, it must be also emphasised that the strongest positive predictor of SVR is not the IL28B CC, but the RVR [30,31] as it was found in our cases too, when 86% of patients with RVR eliminated their HCV1 infections.

The question arises whether HCV1 patients carrying the IL28B TT genotype always require a more complex (e.g. triple) therapy with direct acting antivirals (DAAs)? It is conceivable that patients with the TT homozygosity, who also have other negative predictors (such as previous non-response, high viral load, cirrhosis, obesity, insulin resistance), will be candidates for treatment with DAAs.

Recently, it was found that both the triple therapy with DAAs, (e.g. protease inhibitors plus P/R) and the IFN-free anti-HCV treatment may overcome the effect of the unfavourable TT IL28B polymorphism. Thus, in the future, the relevance of IL28B genotyping may become limited in the individualized treatment for HCV1 infection [32-35]. However, Bronowicki et al. reported that 100% of treatment-naive HCV1 patients with IL28B CC achieved SVR, even with 12 weeks of a protease inhibitor telaprevir and P/R combination therapy [36]. Therefore, IL28B genotyping may continue to be of importance in the shortening of treatment duration.

Thus, the findings of IL28B polymorphisms will allow one to predict with better accuracy which patients are likely to respond to the standard of care P/R therapy. However, it must be stressed that the effect of the favourable variant is not absolute: not all carriers of the “good response genotype” clear the virus; nor do all patients lacking it fail to benefit from the P/R treatment [30,31].

It was noted previously that *IL10 -1082 GG genotype* was frequent in HCV, when mononuclear cells produced two-fold greater quantities of IL-10 compared with patients with IL10 AA genotype [25,26]. Vigidal et al. reported, that IL10 gene promoter −1082 GG genotype occurred more frequently in HCV patients than in controls and that the GG homozygosity was associated with poor response to IFN. This genotype was identified in 34.6% of 80 HCV patients compared with 6/33 (16.7%) of controls (p = 0.048). However, this study focused on limited numbers of both patients and control subjects [28]. Meta-analysis of Zhang et al., also showed that the IL10 -1082 GG genotype was associated with susceptibility for chronic HCV infection [29]. Yet, Afzal et al. reported that the frequency of −1082 GA genotype was higher in healthy individuals than in HCV patients, suggesting the protective effect of this variant [19]. Lio et al. noted that the IL10 -1082 GG was associated with spontaneous recovery from HCV infection [18], while according to Gao et al. -1082 AA conferred an increased risk of persistent HCV infection [37]. Knapp et al. found that HCV patients with SVR showed higher frequency of IL10 -1082 GG genotype than non-responders (26.9% vs 14.0% OR 2.28, 95% CI 1.21-4.32, p = 0.05) [17].

In this study we investigated the IL10R promoter gene polymorphisms at position −1087 relative to transcription start site and proved that the −1087 GG occurs at a lower frequency in HCV patients as compared with controls; thus, this variant may be protective against the disease. At the same time, the frequency of GA genotype and A allele was higher in the patients’ group - as a potential genetic marker of susceptibility to the HCV1 infection. In patients who achieved SVR, the IL10R GG genotype occurred with higher (32.%) frequency than AA (17.4%), and the IL28B CC plus IL10R −1087 A allele combination was also found with higher rate among patients achieving SVR (21.3%), than in non-responders (12.8%).

Our findings on the role of IL10R gene polymorphism in HCV1 infection are rather conflicting, and may contradict both some of the previously published data [28,29] as well as the known biological effect of IL-10. The reasons for these discrepancies are not clear. The study populations may differ with regard to factors that influence SVR: viral load, obesity, fibrosis, ethnicity and sex. Additional studies are required to define the roles of these factors in connection with IL10 polymorphisms in chronic hepatitis C.

## Conclusions

We demonstrated that not only IL28B CC but also IL10R GG genotype and certain IL28B plus IL10R allele combinations may be protective genetic variants in Hungarian patients with chronic HCV1 infection. By predicting which persons are most likely to respond to IFN-based therapy, the examination of these polymorphisms may help to manage an individualized approach to treat HCV patients. Genotyping both IL28B and IL10R may offer a *baseline screening* test, that, however, should be combined with other well-known *pre-* and *on-treatment* predictors for the outcome of the disease and choosing the most appropriate type of anti-HCV therapy. In the era of DAAs for the treatment of HCV infection, the prognostic role of these genetic tests may be of less importance. Yet, in certain circumstances, the IL28B genotyping can be useful; e.g. in the shortening of treatment duration in P/R plus DAA-treated HCV patients. It is evident, however, that as an on-treatment predictor, rapid virological response remains the strongest prognostic factor, even across IL28B genotypes [38].

## Abbreviations

ALT: Alanine aminotransferase; DDA: Direct acting antiviral; GWAS: Genome-wide association study; HCV: Hepatitis C virus; IFN: Interferon; IL: Interleukin; NK: Natural killer; PEG-IFN: Pegylated interferon; P/R: Pegylated interferon plus ribavirin; RBV: Ribavirin; RVR: Rapid virological response; SNP: Single nucleotid polymorphism; SVR: Sustained virological response; Th: T helper; TNF: Tumor necrosis factor.

## Competing interests

The authors declare that they have no competing interests.

## Authors’ contributions

AP, GP, IT, FS, BH initiated and participated in the design of the study, PK carried out the genotyping studies; DV, EF, MP, GL, JF, MV, JG, JS, ZN ZP, AT, AH, ZS, ÁV contributed to acquisition of patients and their data; AP, GP, LS drafted the manuscript; BM revised the paper critically for intellectual content. All authors read and approved the final manuscript.
